# Design Hybrid Porous Organic/Inorganic Polymers Containing Polyhedral Oligomeric Silsesquioxane/Pyrene/Anthracene Moieties as a High-Performance Electrode for Supercapacitor

**DOI:** 10.3390/ijms24032501

**Published:** 2023-01-28

**Authors:** Mohsin Ejaz, Maha Mohammed Samy, Yunsheng Ye, Shiao-Wei Kuo, Mohamed Gamal Mohamed

**Affiliations:** 1 Department of Materials and Optoelectronic Science, College of Semiconductor and Advanced Technology Research, Center for Functional Polymers and Supramolecular Materials, National Sun Yat-Sen University, Kaohsiung 804, Taiwan; 2 Chemistry Department, Faculty of Science, Assiut University, Assiut 71515, Egypt; 3 Department of Medicinal and Applied Chemistry, Kaohsiung Medical University, Kaohsiung 807, Taiwan

**Keywords:** octavinylsilsesquioxane, porous organic–inorganic polymers, heck reaction, thermal stability, energy storage

## Abstract

We synthesized two hybrid organic–inorganic porous polymers (HPP) through the Heck reaction of 9,10 dibromoanthracene (A-Br_2_) or 1,3,6,8-tetrabromopyrene (P-Br_4_)/A-Br_2_ as co-monomers with octavinylsilsesquioxane (OVS), in order to afford OVS-A HPP and OVS-P-A HPP, respectively. The chemical structures of these two hybrid porous polymers were validated through FTIR and solid-state ^13^C and ^29^Si NMR spectroscopy. The thermal stability and porosity of these materials were measured by TGA and N_2_ adsorption/desorption analyses, demonstrating that OVS-A HPP has higher thermal stability (T*_d10_*: 579 °C) and surface area (433 m^2^ g^−1^) than OVS-P-A HPP (T*_d10_*: 377 °C and 98 m^2^ g^−1^) due to its higher cross-linking density. Furthermore, the electrochemical analysis showed that OVS-P-A HPP has a higher specific capacitance (177 F g ^−1^ at 0.5 A F g^−1^) when compared to OVS-A HPP (120 F g ^−1^ at 0.5 A F g^−1^). The electron-rich phenyl rings and Faradaic reaction between the π-conjugated network and anthracene moiety may be attributed to their excellent electrochemical performance of OVS-P-A HPP.

## 1. Introduction

The disparity between the rising energy demand and the stagnant supply has resulted in a worldwide energy crisis [1,2,3,4,5]. The fast production and excessive use of fossil fuels have opened an urgent demand for providing solutions to environmental challenges. Therefore, there is a great need to innovate sustainable and effective energy storage methods [6,7,8,9,10]. Scientists have sought to address this problem by developing renewable energy storage methods. Supercapacitors (SCs) are one of the most practical approaches to addressing energy scarcity [11,12,13], and they have a high energy density, excellent durability, a quick charge/discharge mechanism, and significant stability [14,15,16]. In SCs, energy may be stored in two different ways; the first is the non-faradaic method, in which the ionic charges are gathered electrostatically at the electrolyte–electrode interface. In contrast, in the faradaic method, the activity occurs at the solid surface and involves a reversible redox reaction [17,18]. The electrode material is regarded as one of the main criteria influencing the effectiveness of SCs [19]. Thus, many inorganic, organic, and hybrid organic–inorganic materials have been used as electrode materials for SCs [20,21,22,23,24]. 

Designing new and stable electrode materials is still required to improve SC functionalities. Porous organic polymers (POPs) that have high surface areas, better thermal stability, porous characteristics, and exciting functions have attracted significant attention, leading to their widespread usage in energy storage, optoelectronics, lithium-ion batteries, gas capture, and catalysis [25,26,27,28,29]. These outstanding functionalities of POPs make them attractive candidates as electrode materials for SCs [30,31,32,33,34,35]. POPs are further categorized into conjugated microporous polymers (CMPs), covalent organic polymers (COFs), and hyper-crosslinked polymers (HCPs) [36,37,38,39,40]. Polyhedral oligomeric silsesquioxane (POSS) is suitable for developing porous materials due to its rigid structure and high functionalization [41,42,43,44,45]. The existence of organic–inorganic hybrid components in POSS can provide both organic and inorganic characteristics to develop porous materials [45,46,47,48,49,50]. 

Octavinylsilsesquioxane (OVS) is the most efficient nanosized monomer (1–3 nm) among POSS derivatives due to its accessibility and cheap price [51,52]. OVS-based porous materials can be synthesized using various techniques, including Heck reactions, click reactions, Friedel–Crafts, and hydrosilylation [40,41,42,43,44,45,46,47,48,49,50,51,52,53]. OVS-based POPs materials have an impressive surface area, pore size, thermal stability, and electrochemical performance [54,55,56,57,58]. Furthermore, previous studies have shown that polycyclic aromatic hydrocarbons (PAH) could assist in enhancing energy storage performances [59]. Sandeep et al. presented different PAHs (pyrene, coronene, and triphenylene) as active cathode materials for organic batteries [60]. Kagatikar et al. synthesized pyrene-based chalcones electrode materials for supercapacitors that had a capacitance of 220 F g^−1^ [61]. We synthesized anthracene-based covalent organic frameworks that experienced a specific capacitance of 589 F g^−1^ [62]. Herein, we incorporated polycyclic aromatic hydrocarbons (pyrene and anthracene) into the backbone of OVS to enhance the super-capacitive performance. 

**Scheme 1:**
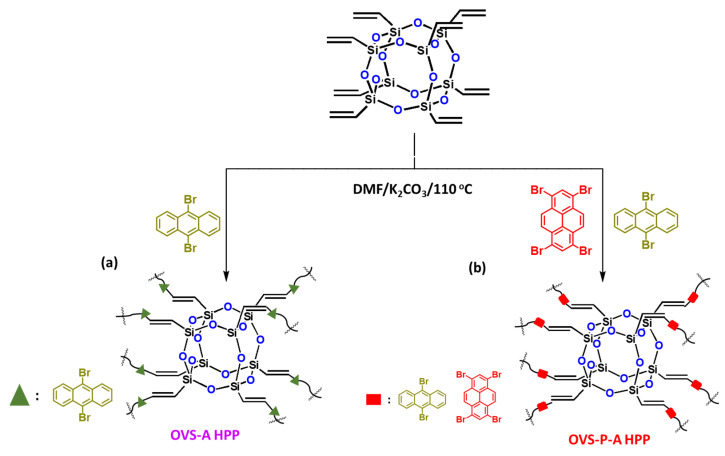
Synthesis of (**a**) OVS-A HPP and (**b**) OVS-P-A HPP through Heck reaction.

In this study, we successfully synthesized two hybrids of a porous polymer-linked OVS unit through a heck reaction of OVS with 9,10-dibromoanthracene (A-Br_2_) and 1,3,6,8-tetrabromopyrene (P-Br_4_)/A-Br_2_ to obtain OVS-A HPP and OVS-P-A HPP, respectively (Scheme 1). Their chemical structures, thermal stability, porosity, morphology, and electrochemical performances are discussed below. 

## 2. Results and Discussion

### 2.1. Characterization, Thermal Stability, Porosity, and Morphology of OVS-A HPP and OVS-P-A HPP

**Figure 1:**
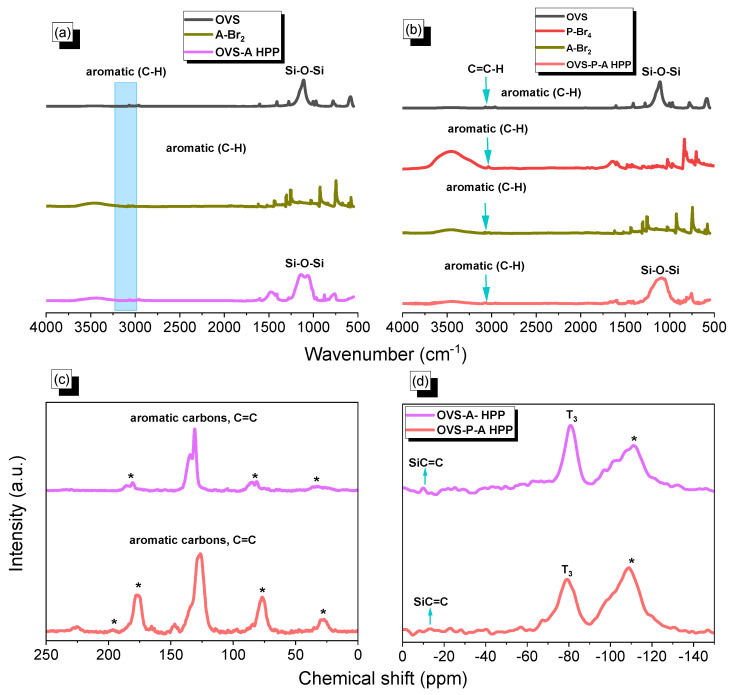
(**a**,**b**) FTIR of OVS, A-Br_2_, P-Br_4_, OVS-A HPP and OVS-P-A HPP, (**c**) ^13^C and (**d**) ^29^Si NMR spectra of the OVS-A HPP and OVS-P-A HPP. * is the side band of solid-state nuclear magnetic resonance spectroscopy (NMR).

The synthesis of two different hybrids of organic–inorganic HPP (OVS-A HPP and OVS-P-A HPP) is shown in Scheme 1. We synthesized A-Br_2_ through the reaction of anthracene and Br_2_ with chloroform at 50 °C for 4 h (Scheme S1). Then, we prepared P-Br_4_ by the reaction of pyrene with a neat Br_2_ solution in nitrobenzene at 120 °C for 24 h (Scheme S2). Finally, we prepared OVS-A HPP and OVS-P-A HPP through the heck reaction of A-Br_2_ and P-Br_4_/A-Br_2_, respectively, with OVS, DMF, Pd(PPh_3_)_4_ and K_2_CO_3_ at 110 °C for 72 h (Scheme 1). All organic solvents showed a low solubility for these OVS-HPPs frameworks (Scheme 1), which is evident in the fact that the Heck reactions were effective, leading to the development of highly crosslinked OVS materials. The chemical structures of OVS-A HPP and OVS-P-A HPP were verified through FTIR and solid-state ^13^C and ^29^Si spectroscopy, as shown in Figure 1. The absorption bands at 3113 cm^−^^1^, 3067 cm^−^^1^, 1610 cm^−^^1^, and 1108 cm^−^^1^ were found in the spectra of OVS, representing the bond stretching of C=CH, C=C, and Si-O-Si, respectively. The absorption band for A-Br_2_, P-Br_4_, OVS-A HPP, and OVS-P-A HPP were observed at 3073 cm^−^^1^, 3031 cm^−^^1^, 3079 cm^−^^1^, and 3065 cm^−^^1^, corresponding to C-H aromatics, respectively (Figure 1a,b). The development of cross-linked networks was also observed in both porous materials (OVS-A HPP, and OVS-P-A HPP), as the absorption spectra of the Si-O-Si unit were wider than that of OVS (Figure 1a,b). Furthermore, due to water absorption, both OVS-A HPP and OVS-P-A HPP featured OH groups in their FTIR spectra. According to the solid-state ^13^C NMR results of the OVS-A HPP and OVS-P-A HPP (Figure 1c), the carbon nuclei signals were found in the range 137–131 ppm and 148–127 ppm, respectively, representing aromatic carbons and C=C groups in both porous OVS-HPP-based materials. Additionally, solid-state ^29^Si NMR analysis was used to verify the functional groups of OVS in the OVS-A HPP and OVS-P-A HPP structures. Figure 1d displays the signals that appeared near −11.2 ppm and −80.2 ppm, corresponding to Si-C=C and T_3_ groups, respectively, for the OVS cage in the OVS-A HPP and OVS-P-A HPP.

**Figure 2:**
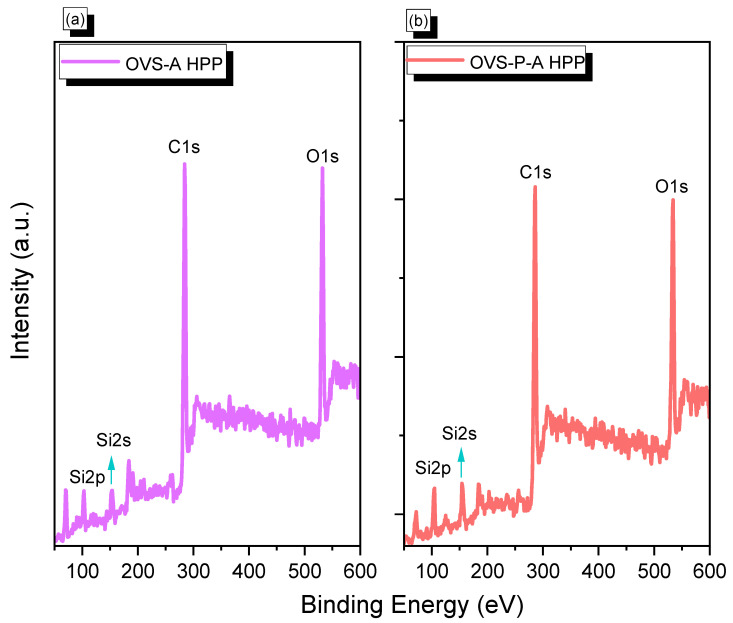
XPS analysis of (**a**) OVS-A HPP and (**b**) OVS-P-A HPP.

We performed X-ray photoelectron spectroscopy (XPS) to confirm the presence of Si, O, and C elements in OVS-A HPP and OVS-P-A HPP (Figure 2). We observed Si2p, Si2s, C1s, and O1s signals corresponding to 103 eV, 155 eV, 284 eV, and 532 eV for OVS-A HPP, respectively (Figure 2a), and 103 eV, 156 eV, 284 eV and 534 eV for OVS-P-A HPP, respectively (Figure 2b). XPS analysis confirmed the successful synthesis of OVS-A HPP and OVS-P-A HPP materials. 

**Figure 3:**
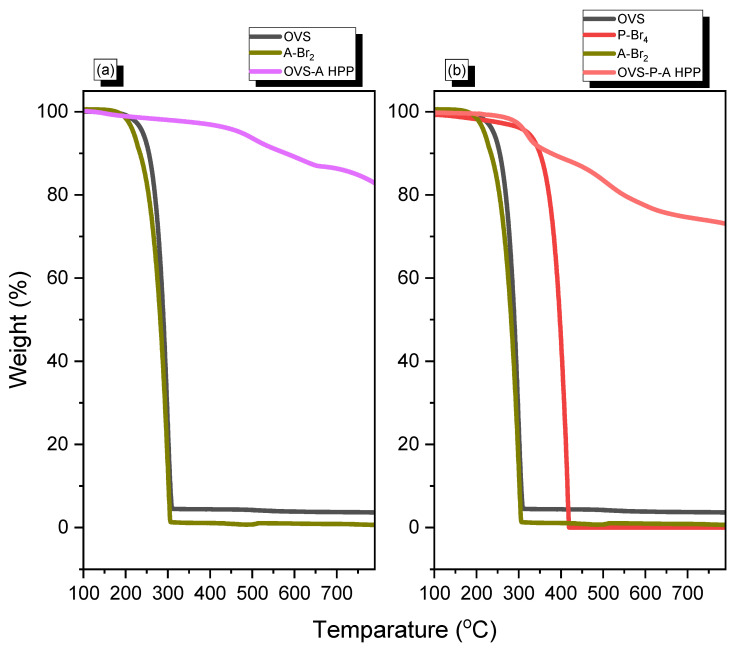
TGA analysis of (**a**) OVS, A-Br_2_, OVS-A HPP and (**b**) OVS, P-Br_4_, A-Br_2_, OVS-P-A HPP.

TGA analysis was used to measure the thermal stability and char yield of OVS-A HPP and OVS-P-A HPP (Figure 3). The thermal decomposition temperatures (T*_d5_*, T*_d10_*) and char yield for OVS were observed as 240 °C, 255 °C, and 4 wt%, respectively. The corresponding values for A-Br_2_ were 219 °C, 234 °C, and 0 wt%, whereas those for P-Br_4_ were 321 °C, 350 °C, and 0 wt%, respectively. We found that the developed hybrid porous material’s thermal stability and char yield were greatly enhanced after the heck reaction, due to the cross-linking of OVS with A-Br_2_ and P-Br_4/_A-Br_2_. The OVS-A HPP experienced a thermal stability of (T*_d5_*, T*_d10_*) 473 °C, 579 °C, and a char yield of 83 wt%; meanwhile, for OVS-P-A HPP, they were 317 °C, 377 °C, and 73 wt%, respectively. The thermal stability and char yield of OVS-A HPP was higher than that of OVS-P-A HPP due to its higher cross-linking density. The thermal properties of two hybrid porous materials are summarized in Table 1. 

**Figure 4:**
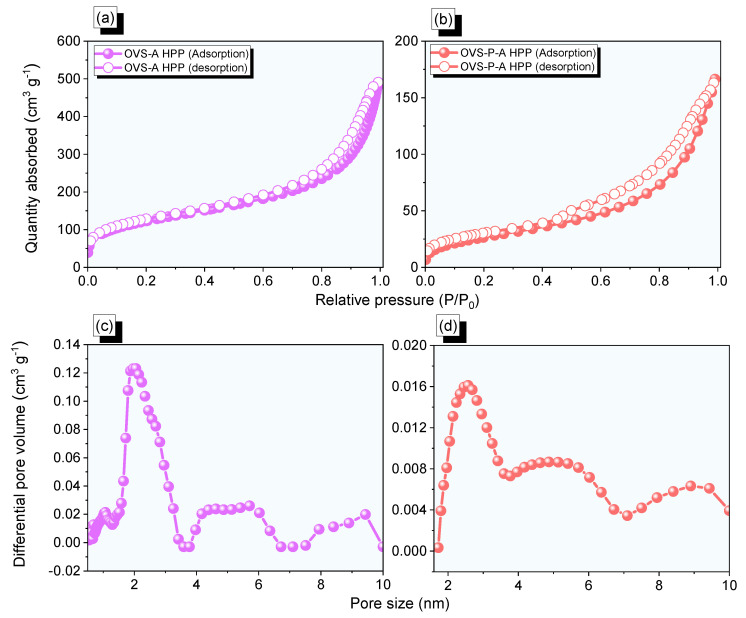
(**a**,**b**) N_2_ isotherms profiles and (**c**,**d**) pore diameter size of the (**a**,**c**) OVS-A HPP and (**b**,**d**) OVS-P-A HPP.

The porosity properties of OVS-A HPP and OVS-P-A HPP were measured by N_2_ adsorption/desorption (Figure 4a,b, and Table 1). The OVS-A HPP exhibited type II adsorption isotherm features, while the OVS-P-A HPP exhibited type II and IV according to the IUPAC classifications. The OVS-A HPP and OVS-P-A HPP exhibited rapid N_2_ absorption uptake potentials in the low and high-pressure zones, indicating the presence of microporous and mesoporous in their structures. Furthermore, the surface area (S_BET_) of OVS-A HPP (433 m^2^ g^−^^1^) was found to be higher than that of OVS-P-A HPP (98 m^2^ g^−^^1^). The OVS-A HPP and OVS-P-A HPP had mean pore diameters of ca. 2 nm and 2.5 nm, respectively, and their total pore volumes were 1.1 cm^3^ g^−^^1^ and 0.3 cm^3^ g^−^^1^, respectively (Figure 4c,d and Table 1).

**Figure 5:**
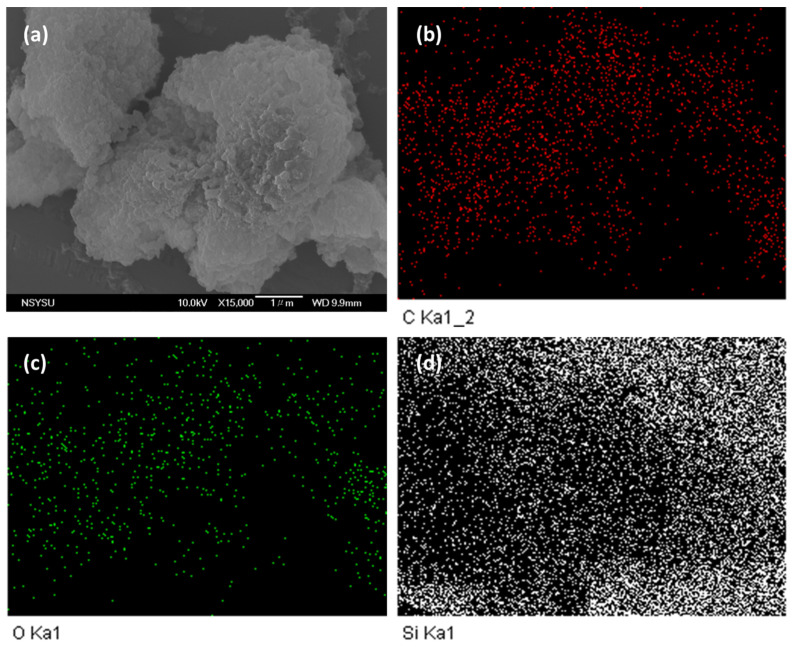
SEM images and their corresponding element mapping for (**a**–**d**) OVS-A HPP.

**Figure 6:**
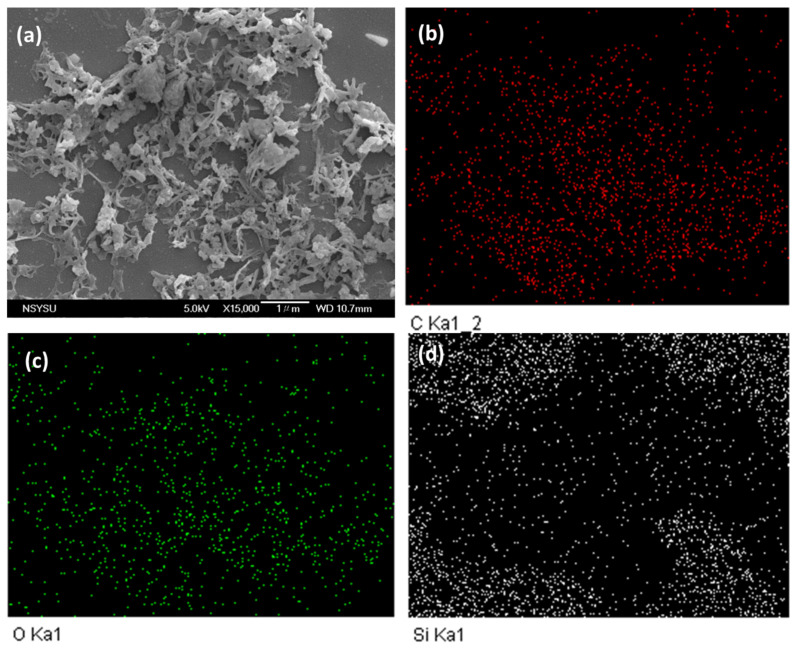
SEM images and their corresponding element mapping for (**a**–**d**) OVS-P-A HPP.

The morphological and porous properties of OVS-A HPP and OVS-P-A HPP were investigated by SEM and TEM (Figure S3). The SEM images of OVS-A HPP showed clustered small spheres (Figure S3a), while for OVS-P-A HPP, we observed clustered irregularly shaped columnar and spheres (Figure S3b). The element mapping and energy dispersive X-ray (EDX) analyses of SEM images confirmed the existence of C, N, and O atoms (Figure 5 and Figure 6). The corresponding weight percentages of C, N, and O atoms were found to be 48.3, 16.1, and 36% for OVS-A HPP, and 33.2, 13.3, and 54% for OVS-P-A HPP. Furthermore, the morphology of hybrid porous materials was further confirmed by the TEM images (Figure S3c,d)). These images show small pores, confirming porous structure, and dark and bright patches representing amorphous characteristics in OVS-A HPP and OVS-P-A HPP.

### 2.2. Electrochemical Properties of OVS-A HPP and OVS-P-A HPP

**Figure 7:**
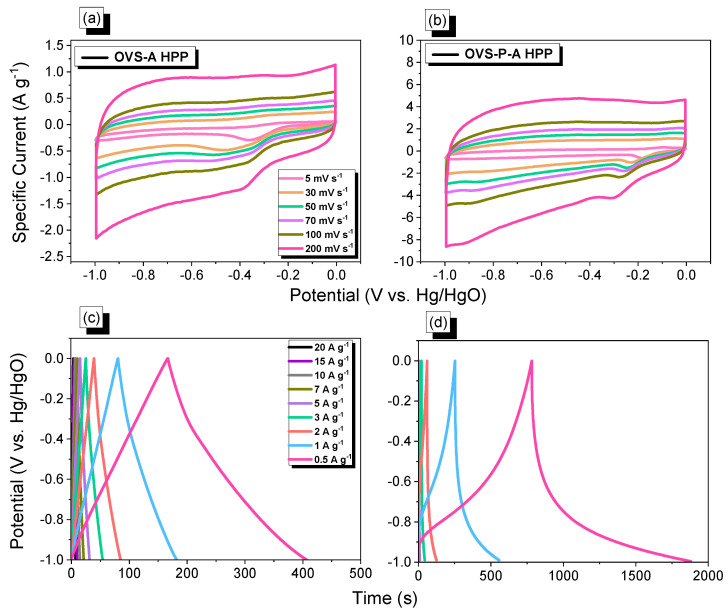
(**a**,**b**) CV and (**c**,**d**) G curves, of (**a**,**c**) OVS-A HPP and (**b**,**d**) OVS-P-A HPP.

The electrochemical properties were investigated in a three-electrode cell (system), using the techniques for cyclic voltammetry (CV) and galvanostatic charge-discharge (GCD) in 1 M of KOH aqueous solution (Figure 7a–d). The CV profiles of OVS-A HPP and OVS-P-A HPP were measured at different scan rates (200 to 5 mV s^−^^1^) and potential windows (−1 to 0 V) (Figure 7a,b). Both the samples experienced rectangular CV curves, with humps suggesting that this capacitive behavior occurred mostly from electric double-layer capacitance (EDLC) and pseudocapacitance [63]. Increasing the scan rate led to a higher specific current without changing the morphologies of the CV profiles, validating the stabilities and the efficient electron mobility [64]. The GCD curves of OVS-A HPP and OVS-P-A HPP were measured at different specific currents (0.5 to 20 A g^−^^1^) (Figure 7c,d). The cathodic peaks can be seen for both electrode materials in CV plots, due to the presence of heteroatoms (O and Si) in the OVS unit and electron-rich phenyl groups in the anthracene and pyrene [53,55]. The GCD curve of OVS-A HPP showed an approximately rectangular curve with a small bend, demonstrating the combined effects of pseudocapacitance and EDLC [65]. The GCD curves of OVS-P-A HPP demonstrated the traditional characteristics of a pseudocapacitance with great symmetry, suggesting a strong electrochemical performance [66]. The discharge time of OVS-P-A HPP was higher and more prominent than OVS-A HPP (Figure 7c,d), showing its comparatively high capacitance. 

**Figure 8:**
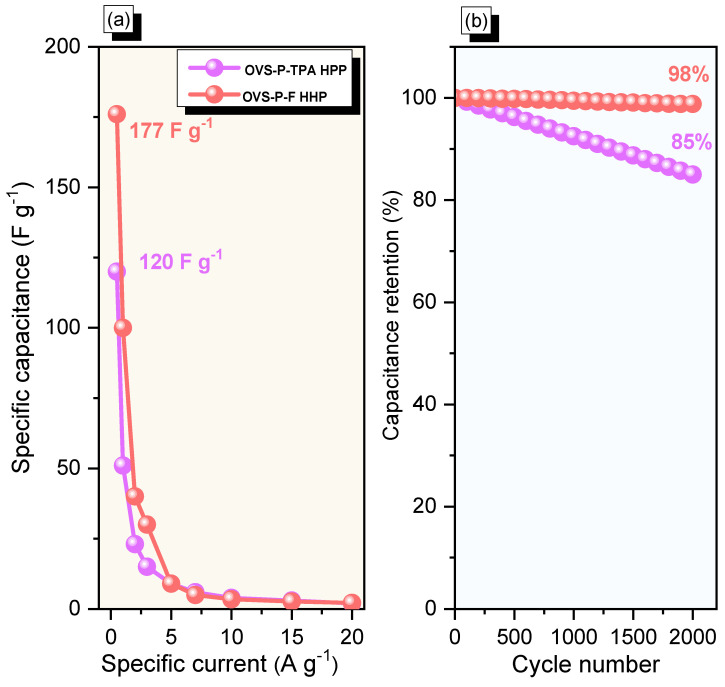
(**a**) Specific capacitance and (**b**) stability profiles for OVS-A HPP and OVS-P-A HPP.

The specific capacitances of OVS-A HPP and OVS-P-A HPP, calculated from GCD curves, were 120 F g^−^^1^ (calculated by using Equation (S1)) and 177 F g^–1^ (calculated by using Equation (S2)), respectively, at 0.5 A g^−^^1^ (Figure 8a). The difference in the specific capacitance between the OVS-A HPP and OVS-P-A HPP was very pronounced when the specific current was increased to 20 A g^−^^1^, experiencing the values of 2 F g^–1^ and 2.5 F g^–1^, respectively. As a result, the overall CV and GCD analysis showed that OVS-P-A HPP showed higher electrochemical properties than OVS-A HPP. The chemical structure of OVS-P-A HPP contains pyrene groups with more electron-rich phenyl rings, allowing the electrolytes to reach the electrode surface more quickly than in the OVS-A HPP; this was responsible for its comparatively remarkable performance [67]. The Faradaic reaction between anthracene and the π-conjugated framework may also be responsible for OVS-P-A HPP’s excellent performance. When the specific current was increased from 0.5 to 20 A g^−^^1^, the specific capacitance of OVS-A HPP and OVS-P-A HPP declined, most likely because there was not enough time for ion mobility at such high specific currents [68]. The specific capacitance retention of OVS-A HPP and OVS-P-A HPP was also measured at 2000 cycles from GCD analysis (Figure 8b). The OVS-A HPP and OVS-P-A HPP exhibited excellent stability, with a specific capacitance retention of 85% and 98%, respectively. Additionally, the electrochemical performance of OVS-A HPP and OVS-P-A HPP was exceptional when compared to previously reported porous polymers and composite materials (Table S1) [53,69,70,71,72,73,74,75].

In addition, we examined the electrochemical properties of the OVS-A HPP and OVS-P-A HPP for a symmetric supercapacitor using coin cells (Figure S4). The CV profiles of OVS-A HPP and OVS-P-A HPP were measured at the same scan rates and potential windows as the three electrodes (Figure S4a,b). The CV curve for OVS-A HPP (Figure S4a) is quite similar to the three-electrode system, but in OVS-P-A HPP (Figure S4b), the humps appeared more prominently to assist in the presence of both EDLC and pseudocapacitance. The pure triangular GCD curve was observed for OVS-A HPP, suggesting the presence of EDLC (Figure S4c), and OVS-P-A HPP experienced a triangular curve with some bends demonstrating EDLC and a pseudocapacitive response (Figure S4d). The specific capacity was observed as 33 F g^−^^1^ and 80 F g^−^^1^ for OVS-A HPP and OVS-P-A at 0.5 A g^−^^1^, respectively. Therefore, two and three electrodes revealed the significant electrochemical performance of OVS-A HPP and OVS-P-A HPP. Based on the two-electrode system, the OVS-P-A HPP had a better energy density (40 Wh Kg^−^^1^) than the OVS-A HPP (16 Wh Kg^−^^1^) (Figure S5). 

## 3. Materials and Methods

### 3.1. Materials

We ordered potassium carbonate (K_2_CO_3_), nitrobenzene (C_6_H_5_NO_2_), bromine solution (Br_2_), and octavinylsilsesquioxane (OVS) from Alfa Aesar. Acros Organics provided acetone, methanol (MeOH), chloroform (CHCl_3_), and tetrahydrofuran (THF), as well as N,N-dimethylformamide (DMF), and methanol (MeOH). Anthracene (A), pyrene (P), and tetrakis (triphenylphosphine) palladium (0) [Pd(PPh_3_)] were supplied from Sigma-Aldrich. The instrumental methods and electrochemical experimental conditions can be found in the Supplementary File.

### 3.2. Synthesis of 9,10-Dibromoanthracene (A-Br_2_)

Anthracene (5.00 g, 2.80 mmol), Br_2_ (12.5 mL), and CHCl_3_ were added to a two-neck flask and heated at 60 °C in an N_2_ environment for 5 h. After that, the yellow solid (A-Br_2_, 85%) was filtered. ^1^H-NMR (500 MHz, CDCl_3_, Figure S1): 8.6 (s, 2H), 7.6 (s, 2H). ^13^C-NMR (124 MHz, CDCl_3_, Figure S2): 133, 128, 127, 123.

### 3.3. Synthesis of 1,3,6,8-Tetrabromopyrene (P-Br_4_)

Pyrene (6.00 g, 30 mmol), Br_2_ (12 mL), and C_6_H_5_NO_2_ (200 mL) were added to a two-neck flask and heated at 120 °C in an N_2_ environment for 24 h; after that, the yellow solid (P-Br_4_, 91%) was filtered and washed by EtOH and dried under reduced pressure before it was used in the reaction. The NMR data of P-Br_4_ were not provided in this work due to its poor solubility in all organic solvents.

### 3.4. Synthesis of OVS-A HPP and OVS-P-A HPP

OVS (0.40 g, 0.63 mmol), A-Br_2_ (0.85 g, 2.53 mmol), [P-Br_4_ (0.003 g, 0.064 mmol)/A-Br_2_ (0.81 g, 2.41 mmol)], K_2_CO_3_ (3 g) and Pd(PPh_3_)_4_ (0.08 g) in DMF (30 mL), were added to the two-neck flask, and the reaction mixture was refluxed for 72 h at 110 ℃. Then, the resulting solid was washed with different solvents (THF, methanol, and acetone) to obtain a green powder (0.38 g, 95%) for OVS-A HPP or a yellow powder (0.30 g, 75%) for OVS-P-A HPP. The instrumental methods and experimental conditions can be found in the Supplementary File.

## 4. Conclusions

Two different hybrid porous polymers of OVS-A HPP and OVS-P-A HPP were successfully synthesized through the heck reaction of OVS with anthracene and pyrene/anthracene. N_2_ isothermal profiles showed the presence of micro and mesoporous properties in them with BET surfaces of 433 m^2^ g^−1^ and 98 m^2^ g^−1^ for OVS-A HPP and OVS-P-A HPP, respectively. The TGA analysis revealed that OVS-A HPP experienced a higher thermal stability and char yield (579 °C and 83 wt%) than OVS-P-A HPP (377 ^o^C and 73 wt%) due to the higher cross-linking density of anthracene with OVS. Furthermore, the specific capacitance of OVS-A HPP and OVS-P-A HPP was 120 F g^−1^ and 177 F g^−1^, respectively. The electrochemical analysis demonstrated that OVS-P-A HPP exhibited higher super-capacitive performance than OVS-A HPP and the other reported porous polymer materials. 

## Figures and Tables

**Table 1:** Thermal stability, porosity, and electrochemical performance of OVS-A HPP and OVS-P-A HPP.

Sample	T*_d10_* (°C)	Char Yield (wt.%)	Surface Area (m^2^/g)	Pore Volume (cm^3^/g)	Pore Size (nm)	Specific Capacitance at 0.5 A g^−1^ (F g^−1^)
OVS-A HPP	579	83	433	1.1	2	120
OVS-P-A HPP	376	73	98	0.3	2.5	177

## Data Availability

The data presented in this study are available on request from the corresponding author.

## Supplementary Materials

The supporting information can be downloaded at: https://www.mdpi.com/article/10.3390/ijms24032501/s1.
