# Supplementary material for: Design Hybrid Porous Organic/Inorganic Polymers Containing Polyhedral Oligomeric Silsesquioxane/Pyrene/Anthracene Moieties as a High-Performance Electrode for Supercapacitor
Source: Int J Mol Sci. 2023 Jan 28;24(3):2501. doi: 10.3390/ijms24032501 (PMC9916954; doi:10.3390/ijms24032501)
Supplement: Supplementary file 1 [file ijms-24-02501-s001.zip › ijms-2151827-supplementary.pdf]

Supporting Information for

Design Hybrid Porous Organic/Inorganic Polymers Containing Polyhedral Oligomeric Silsesquioxane/Pyrene/Anthracene Moieties as a High-Performance Electrode for Supercapacitors

Mohsin Ejaz[1], Maha Mohammed Samy[1,2], Yunsheng Ye[1], Shiao-Wei Kuo[1,3*] and Mohamed Gamal Mohamed[1,2,*]

Characterization

FTIR spectra were collected using a Bruker Tensor 27 FTIR spectrophotometer at a resolution of 4 cm^{-1} and the KBr disk method. ^{13}C nuclear magnetic resonance (NMR) spectra were recorded using an INOVA 500 instrument, with $CDCl_3$ as the solvent and tetramethylsilane (TMS) as the external standard; chemical shifts are reported in parts per million (ppm). The thermal stabilities of the samples under N_2 were measured using a TG Q-50 thermogravimetric analyzer; the cured sample (ca. 5 mg) was placed in a Pt cell and then heated at 20 °C min^{-1} from 100 to 800 °C under a N_2 flow of 60 mL min^{-1}. Wide-angle X-ray diffraction (WAXD) patterns were measured at the wiggler beamline BL17A1 of the National Synchrotron Radiation Research Center (NSRRC), Taiwan; a triangular bent Si (111) single crystal was used to obtain a monochromated beam having a wavelength (λ) of 1.33 Å. The morphologies of the polymer samples were examined through field emission scanning electron microscopy (FE-SEM; JEOL JSM7610F) and transmission electron microscopy (TEM), using a JEOL-2100 microscope operated at an accelerating voltage of 200 kV. BET surface areas and porosimetry measurements of the samples (ca. 40–100 mg) were performed using a BEL Master^{TM} instrument and BEL sim^{TM} software (v. 3.0.0); N_2 adsorption and desorption isotherms were generated through incremental exposure to ultrahigh-purity N_2 (up to ca. 1 atm) in a liquid N_2 (77 K) bath; surface parameters were calculated using the BET adsorption models in the instrument's software. The pore sizes of the prepared samples were determined using nonlocal density functional theory (NLDFT).

Electrochemical Analysis

Working Electrode Cleaning: Prior to use, the glassy carbon electrode (GCE) was polished several times with 0.05-μm alumina powder, washed with EtOH after each polishing step, cleaned through sonication (5 min) in a water bath, washed with EtOH, and then dried in air.

Electrochemical Characterization: The electrochemical experiments were performed in a three-electrode cell using an Autolab potentiostat (PGSTAT204) and 1 M KOH as the aqueous electrolyte.

The GCE was used as the working electrode (diameter: 5.61 mm; 0.2475 cm^2); a Pt wire was used as the counter electrode; Hg/HgO (RE-1B, BAS) was the reference electrode. All reported potentials refer to the Hg/HgO potential. A slurry was prepared by dispersing the OVS-A HPP or OVS-P-A HPP sample (45 wt. %), carbon black (45 wt. %), and Nafion (10 wt. %) in a mixture of (EtOH/ H_2O) (200 μL: 800 μL) and then sonicated for 1 h. A portion of this slurry (10 μL) was pipetted onto the tip of the electrode, which was then dried in the oven for 30 min at 40 °C. The electrochemical performance was studied through CV at various sweep rates (5–200 mV s^{-1}) and through the GCD method in the potential range from 0 to –1.00 V (vs. Hg/HgO) at various specific currents (0.5–20 A g^{-1}) in 1 M KOH as the aqueous electrolyte solution.

The specific capacitance was calculated from the GCD data using the equation:

$$C_s = (I\Delta t)/(m\Delta V) \qquad \textbf{(S1)}$$

Where C_s (F g^{-1}) is the specific capacitance of the supercapacitor, I (A) is the discharge current, ΔV (V) is the potential window, Δt (s) is the discharge time, and m (g) is the mass of the NPC on the electrode. The energy density (E, W h kg^{-1}) and power density (P, W kg^{-1}) were calculated using the equations.

$$E = 1000C(\Delta V)^2/(2 \times 3600)$$

$$P = E/(t/3600)$$

For capacitance calculation for the non-linear GCD curve for the OVS-P-A HPP sample (**Figure 6(d)**):

$$C = \frac{2I\int V dt}{mV^2} \qquad \textbf{(S2)}$$

Where: $\int V dt$ is the area under the discharge curve obtained from GCD; $\frac{I}{m}$ is the specific current (0.5-20 A/g); and V is the potential window.

We evaluated the electrochemical functionality of a symmetric supercapacitor using a CR2032 coin cell, which consists of an anode and cathode, a bottom and top cover, a metal spring, a separator, and an electrolyte. Our compounds served as both the cathode and the anode in order to construct a symmetric supercapacitor. The slurry was created by combining 2 mg of OVS-A HPP or OVS-A-P HPP, 2 mg of conductive carbon, 20 mL of Nafion, 200 mL of ethanol, and 400 mL of water. It was then sonicated for an hour and cast onto carbon paper. We used a Selemion AMV membrane with an electrolyte of 1.0 M aqueous KOH.

The specific capacitance was calculated in assembled supercapacitor from the GCD data using the following equations:

$C_s = 2(I\Delta t)/(m\Delta V)$ **(S3)**

where C_s (F g^{-1}) is the specific capacitance of the supercapacitor, I (A) is the discharge current, ΔV (V) is the potential window, Δt (s) is the discharge time, and m (g) is the mass of the CMP in the single electrode.

The energy density (E, W h kg^{-1}) and power density (P, W kg^{-1}) were calculated using the equations:

$E_{cell} = 1000\ C_s\ (\Delta V)^2/(4.2.3600)$ **(S4)**

and

$P_{cell} = E_{cell}\ /(t/3600)$ **(S5)**

Table S1. Comparison between the specific surface area/specific capacitance OVS-A HPP and OVS-P-A HPP with those of previously reported materials for supercapacitor application.

Electrode	S_{BET} (m^2 g^{-1})	Capacitance	Ref.
**OVS-A HPP**	433	120 F g^{-1} at 0.5 A g^{-1}	This work
**OVS-P-A HPP**	98	177 F g^{-1} at 0.5 A g^{-1}	This work
**POSS-OXD POIP**	94	119 F g^{-1} at 0.5 A g^{-1}	S1
**POSS-Th POIP**	605	213 F g^{-1} at 0.5 A g^{-1}	S1
**POSS-TPTh POIP**	682	354 F g^{-1} at 0.5 A g^{-1}	S1
**Cz-Cz CMP**	623	43.70 F g^{-1} at 0.5 A g^{-1}	S2
**Cz-TPA CMP**	618	271.82 F g^{-1} at 0.5 A g^{-1}	S2
**Cz-TP CMP**	412	67.38 F g^{-1} at 1 A g^{-1}	S2
**POSS-A-POIP**	426	152.5 F g^{-1} at 0.5 A g^{-1}	S3
**POSS-F-POIP**	452	36.2 F g^{-1} at 0.5 A g^{-1}	S3
**H-THAQ**	-	15 F g^{-1} at 1 A g^{-1}	S4
**THAQ/rGO (2:1)**	-	76 F g^{-1} at 1 A g^{-1}	S4
**Pure AQ**	-	42 F g^{-1} at 1 A g^{-1}	S5
**Fc-CMPs**	653.2	147 F g^{-1} at 0.5 A g^{-1}	S6
**An-CPOP-1**	580	72.75 F g^{-1} at 0.5 A g^{-1}	S7
**An-CPOP-2**	1130	98.4 F g^{-1} at 0.5 A g^{-1}	S7
**DPT-HPP**	1230	110 F g^{-1} at 0.5 A g^{-1}	S8
**TAT-CMP-1**	88	141 F g^{-1} at 1 A g^{-1}	S9
**TPE-DDSQ-POIP**	157.67	22 F g^{-1} at 1 A g^{-1}	S10
**Car-DDSQ-POIP**	256.34	23 F g^{-1} at 1 A g^{-1}	S10

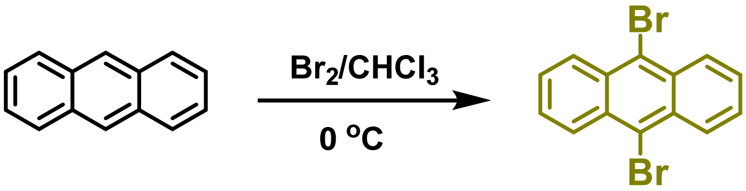

Scheme S1. Synthetic route for the preparation of A-Br_2

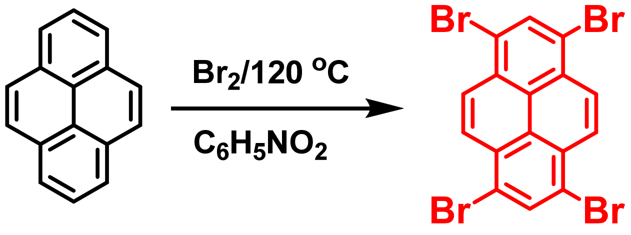

Scheme S2. Synthetic routes for the preparation of P-Br4.

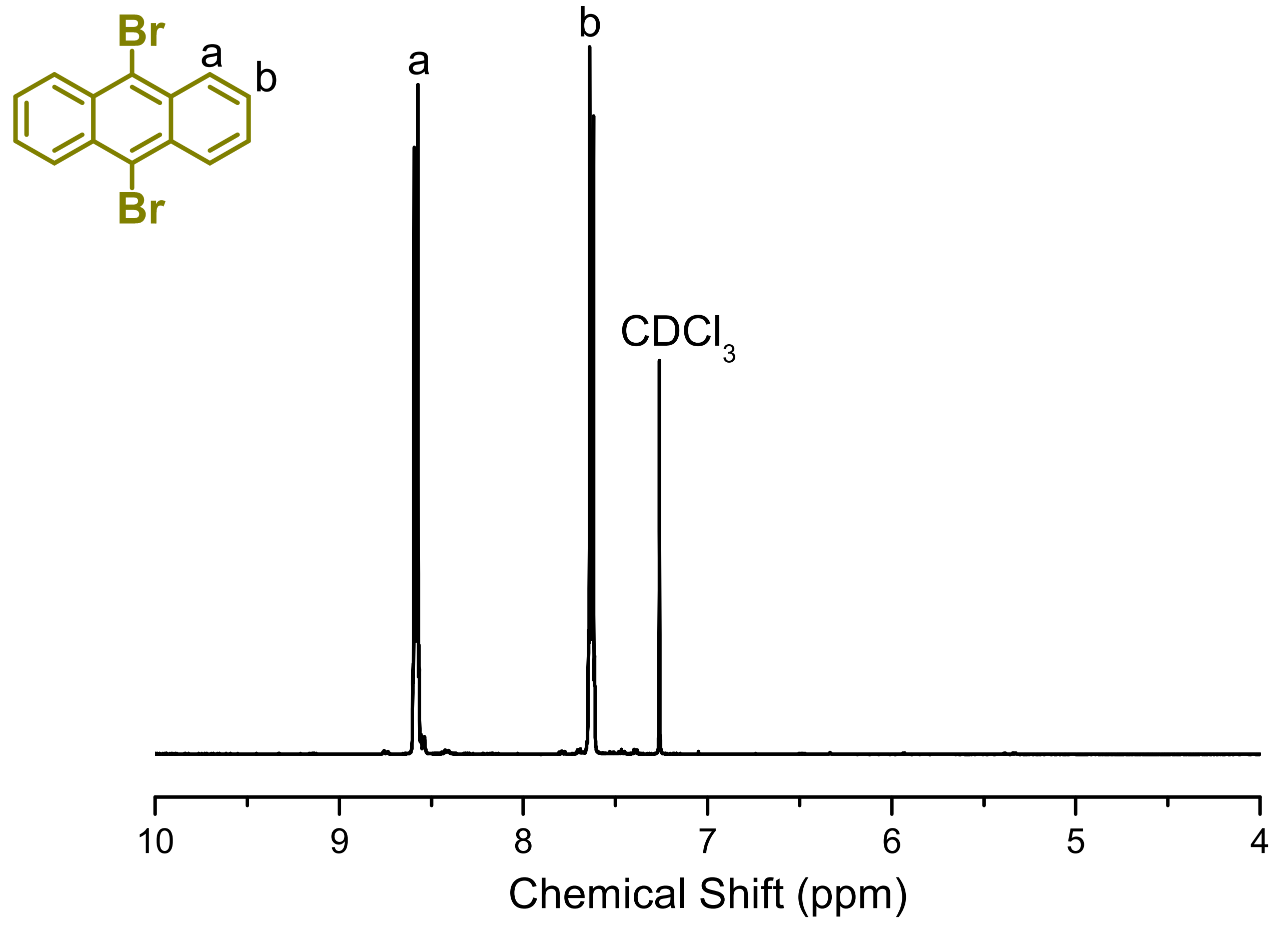

Figure S1. ^{1}H-NMR spectrum of A-Br_2.

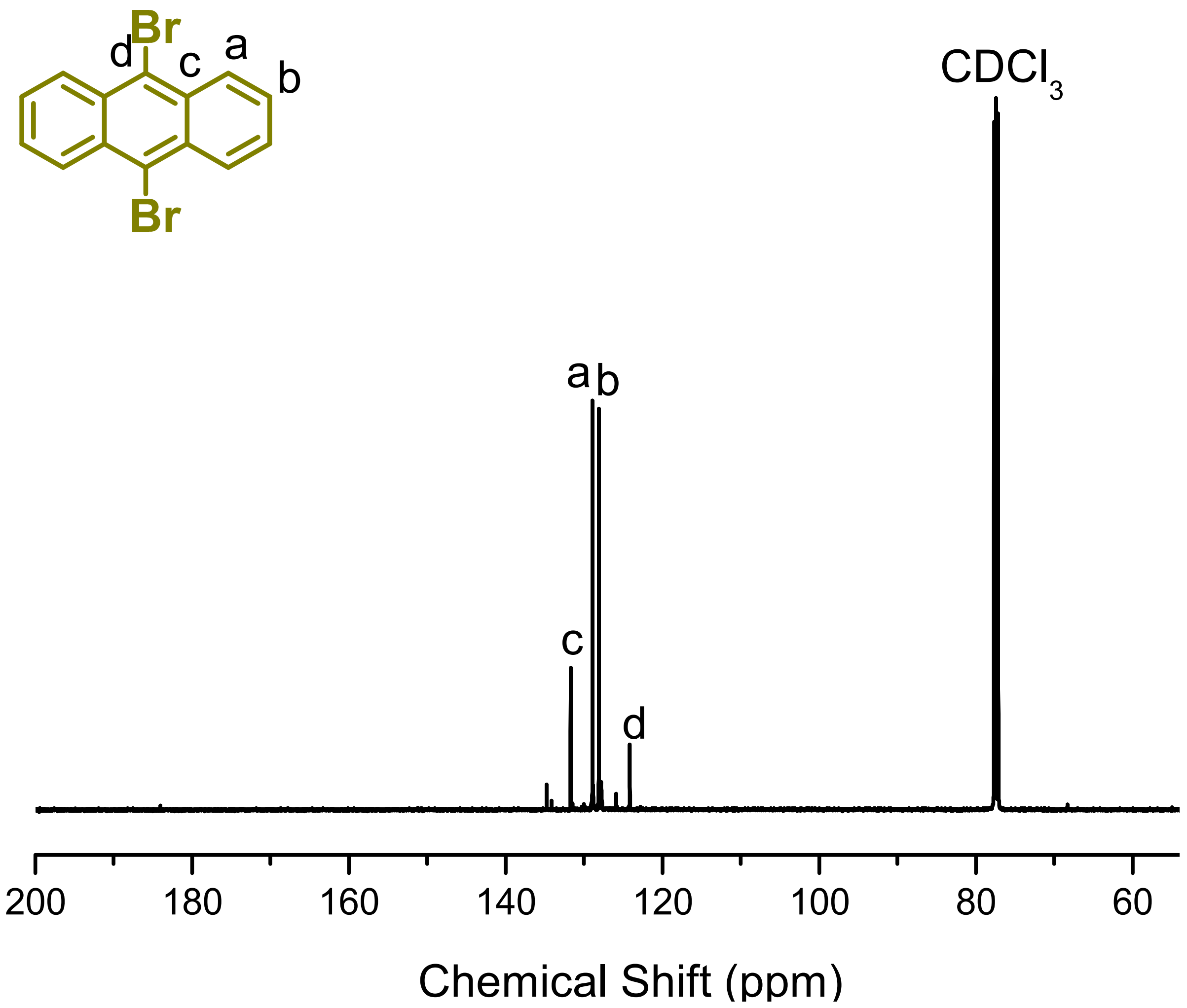

Figure S2. ^{13}C-NMR spectrum of A-Br$_2$.

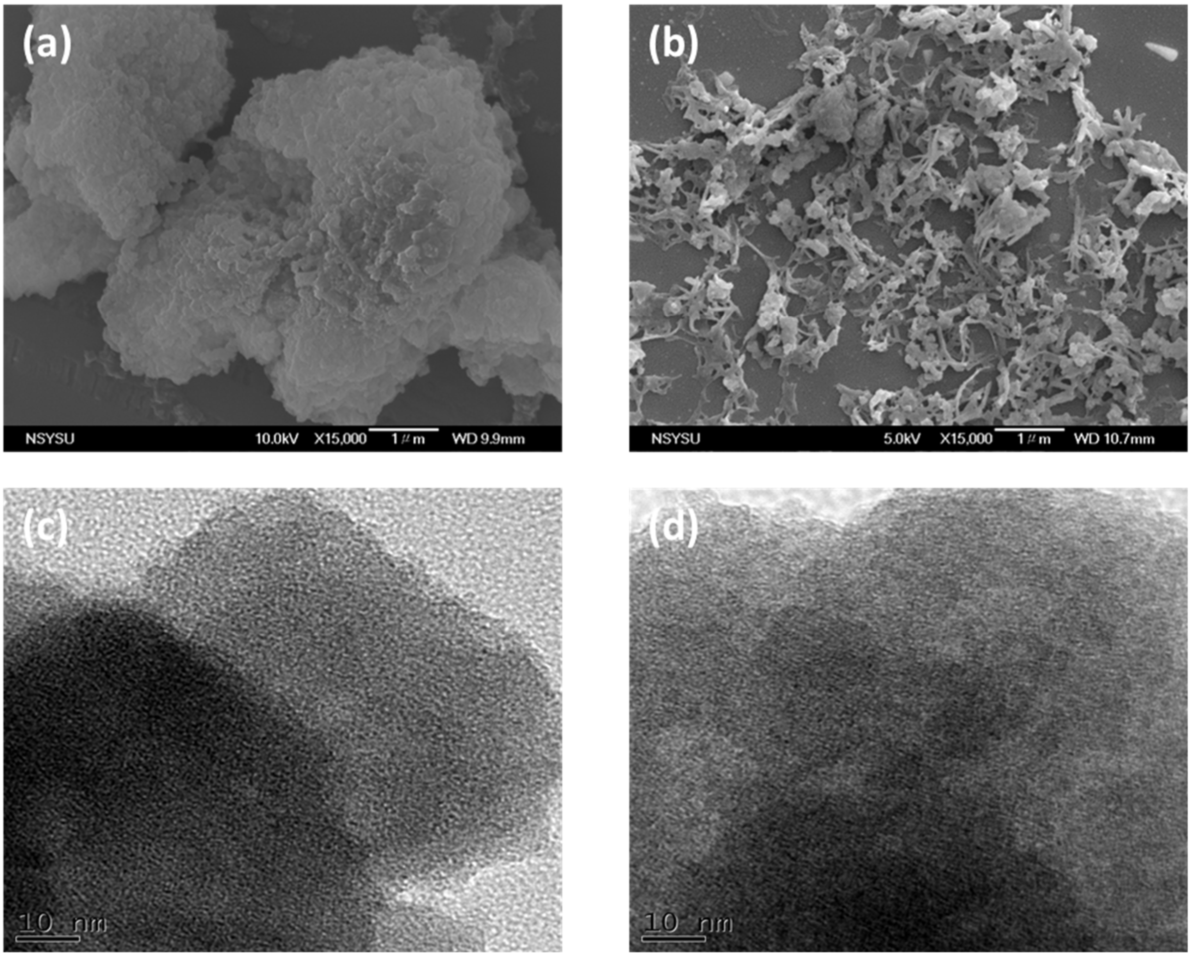

Figure S3. (a–b) SEM and (c-d) TEM images of the (a, c) OVS-A HPP, (b, d) OVS-P-A HPP.

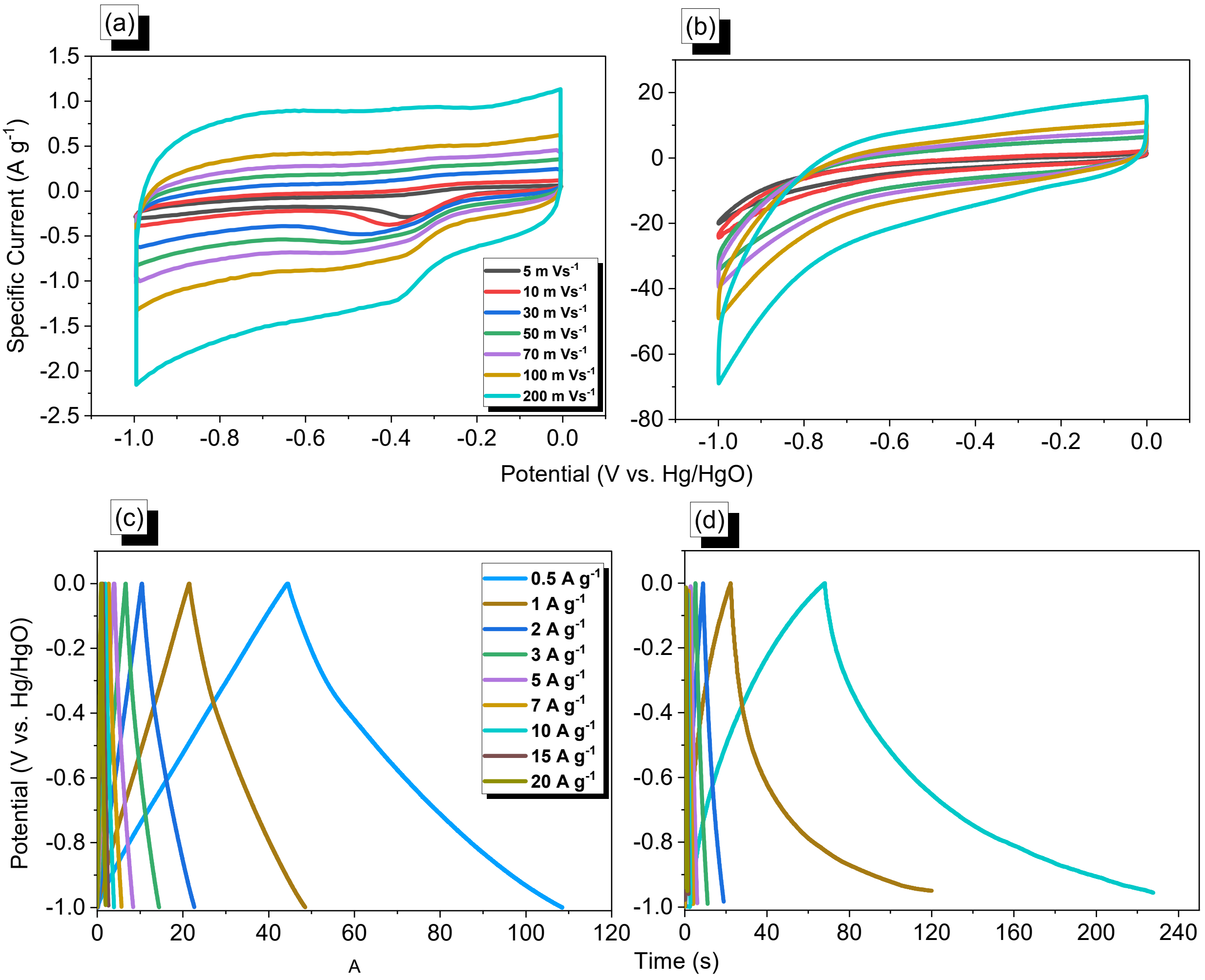

Figure S4. (a, b) CV and (c, d) GCD curves of SSC coin cells incorporating the (a, c) OVS-A HPP and (b, d) OVS-P-A HPP.

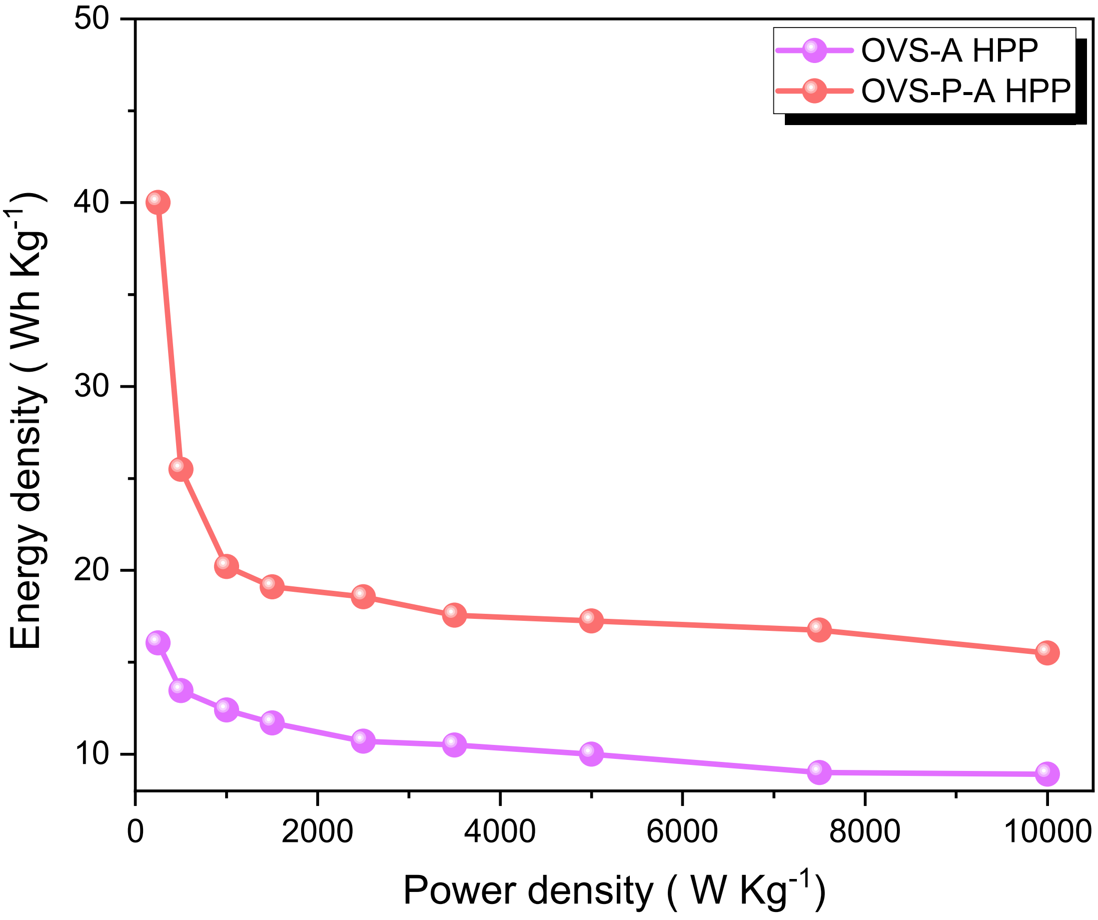

Figure S5. Ragone profiles of OVS-A HPP and OVS-P-A HPP were derived from the two-electrode system.

References

[S1] Ejaz, M.; Mohamed, M. G.; Sharma, S. U.; Lee, J. T.; Huang, C. F.; Chen, T.; & Kuo, S. W. An Ultrastable Porous Polyhedral Oligomeric Silsesquioxane/Tetraphenylthiophene Hybrid as a High-Performance Electrode for Supercapacitors. *Molecules*, (2022) 27(19), 6238. doi.org/10.3390/molecules27196238

[S2] Saber, A. F.; Sharma, S. U.; Lee, J. T.; EL-Mahdy, A. F.; & Kuo, S. W . Carbazole-conjugated microporous polymers from Suzuki–Miyaura coupling for supercapacitors. *Polymer*, (**2022**) 254, 125070. doi.org/10.1016/j.polymer.2022.125070.

[S3] Mohamed, M.G.; Mansoure, T.H.; Takashi, Y.; Samy, M.M.; Chen, T.; Kuo, S.-W. Ultrastable porous organic/inorganic polymers based on polyhedral oligomeric silsesquioxane (POSS) hybrids exhibiting high performance for thermal property and energy storage. *Microporous Mesoporous Mater.* 2021, 328, 111505. doi.org/10.1016/j.micromeso.2021.111505.

[S4] Xu, L.; Shi, R.; Li, H.; Han, C.; Wu, M.; Wong, C.P. Kang, F.; Li, B. Pseudocapacitive anthraquinone modified with reduced graphene oxide for flexible symmetric all-solid-state supercapacitors, *Carbon*, 127 (2018) 459-468. doi.org/10.1016/j.carbon.2017.11.003.

[S5] Guo, B.; Yang, Y.; Hu, Z.; An, Y.; Zhang, Q.; Yang, X.; Wang, X.; Wu, H. Redox-active organic molecules functionalized nitrogen-doped porous carbon derived from metal-organic framework as electrode materials for supercapacitor, *Electrochim. Acta,* 223 (2017) 74–84. doi.org/10.1016/j.electacta.2016.12.012.

[S6] Khattak, A. M.; Sin, H.; Ghazi, Z.A.; He, X.; Liang, B.; Khan, N.A.; Alanagh, H.R.; Iqbal, A.; Li, L.; Tang, Z. Controllable fabrication of redox-active conjugated microporous polymers on reduced graphene oxide for high performance faradaic energy storage, *J. Mater. Chem. A,* 6 (2018)18827-18832. doi.org/10.1039/C8TA07913G.

[S7] Mohamed, M.G.; Zhang, X.; Mansoure, T.H.; Mahdy, A.F.M. EL.; Huang, C.F.; Danko, M.; Xin, Z.; Kuo, S.W. Hypercrosslinked porous organic polymers based on tetraphenylanthraquinone for

CO_2 uptake and high-performance supercapacitor, *Polymer*, 205 (2020) 122857-122866. doi.org/10.1016/j.polymer.2020.122857

[S8] Mohamed, M.G.; Mahdy, A.F.M. EL.; Meng, T.S.; Samy, M.M.; Kuo, S.W. Multifunctional Hypercrosslinked Porous Organic Polymers Based on Tetraphenylethene and Triphenylamine Derivatives for High-Performance Dye Adsorption and Supercapacitor, *Polymers* 12 (2020) 2426. doi.org/10.3390/polym12102426.

[S9] Li, X.C.; Zhang, Y.; Wang, C. Y.; Y. Wan, Lai, W.Y.; Pang, H.; Huang, W. Redox-active triazatruxene-based conjugated microporous polymers for high-performance supercapacitors. *Chem. Sci.,* 8 (2017) 2959-2965. doi.org/10.1039/C6SC05532J.

[S10] Mohamed, M.G.; Chen, W.C; Mahdy, A.F.M.; Kuo, S.W. Porous organic/inorganic polymers based on double-decker silsesquioxane for high-performance energy storage*, J. Polym. Res.* 28 (2021) 219. doi.org/10.1007/s10965-021-02579-x
